# Language, Memory, and Mental Time Travel: An Evolutionary Perspective

**DOI:** 10.3389/fnhum.2019.00217

**Published:** 2019-07-04

**Authors:** Michael C. Corballis

**Affiliations:** School of Psychology, Faculty of Science, University of Auckland, Auckland, New Zealand

**Keywords:** displacement, evolution, externalization, gesture, imagination, memory, mental time travel, universal grammar

## Abstract

Language could not exist without memory, in all its forms: working memory for sequential production and understanding, implicit memory for grammatical rules, semantic memory for knowledge, and episodic memory for communicating personal experience. Episodic memory is part of a more general capacity for mental travel both forward and backward in time, and extending even into fantasy and stories. I argue that the generativity of mental time travel underlies the generativity of language itself, and could be the basis of what Chomsky calls I-language, or universal grammar (UG), a capacity for recursive thought independent of communicative language itself. Whereas Chomsky proposed that I-language evolved in a single step well after the emergence of *Homo sapiens*, I suggest that generative imagination, extended in space and time, has a long evolutionary history, and that it was the capacity to share internal thoughts, rather than the nature of the thoughts themselves, that more clearly distinguishes humans from other species.

## Introduction

Memory, in all its forms, is critical to language. Because language is sequential, we need short-term memory (working memory) as a moving window of consciousness if we are to integrate over time to make sense of sentences, and indeed stories. Long-term memory is itself divided into several components, each also serving a necessary function in linguistic communication. First is the distinction between unconscious and conscious memory. The rules of language are in large part overlearned and unconscious, and even linguists have not completely articulated how those rules work. They operate largely automatically; we know intuitively how to construct a sentence, but do not really know how we do it. Conscious memory is sometimes also referred to as declarative memory, or memory that can be declared. If part of memory is declarative memory, so part of language is memorial declaration.

Conscious memory can, in turn, be divided into semantic memory, or basic knowledge, and episodic memory, which is memory for personal episodes. Broadly speaking, semantic memory is a combined internal dictionary and encyclopedia, while episodic memory is an internal diary that records personal experiences (Tulving, 1972). Language draws on both. Semantic memory includes the large data bank of the tens of thousands of words that we use to express our thoughts, as well as providing kinds of knowledge that we can and do talk about—the political situation, the history of Ireland, differential calculus. It is episodic memory, though, that gives language many of its most distinctive properties.

Episodic memory is part of the more general capacity for *mental time travel*, a term probably first used by Tulving (1985) and elaborated by (Suddendorf and Corballis, 1997, 2007) We can travel mentally into a personal future as well as a personal past, and even create purely fictional events that need have no reference to specific time (“Once upon a time”). These are constructive acts—even episodic memory itself is better regarded as a construction than as a replay, and not always accurate. As Neisser (2008) put it, “Remembering is not like playing back a tape or looking at a picture; it is more like telling a story” (p. 88). Mental time travel is in turn founded on the understanding of space and time, with events encoded according to *what* happened, *where* it happened, and *when* it happened (the www criterion; Suddendorf and Corballis, 2007). I argue in this article that mental time travel provides the basis for the generative and creative aspects of language, allowing us to communicate about past and future, and indeed tell stories that need have no basis in reality.

Language, whether spoken or signed, can then be considered a device by which we share our mental travels—as Dor (2015) put it, it allows “the instruction of imagination.” Indeed, the recursive, generative nature of language may itself derive, not from the structure of language itself, but from the structure of the imaginative thoughts that underlie it.

## Mental Time Travel and Universal Grammar

This view has some connection to the approach to language known as the Minimalist Program (Chomsky, 1995, 2015), but it also differs in important ways. A central tenet of the Minimalist Program is that language is structured by *universal grammar* (UG), which is common to all peoples. UG is the primary component of *I-language*, where the “I” is taken to suggest “internal,” “individual,” and “intensional.” Its main property is *merge*, a recursive operation that allows elements to be combined, and the mergers themselves to be merged, in a progressive fashion to build structures of any desired degree of complexity. The notion of UG has been criticized on the grounds that the 6,000 or so languages of the world have diverse grammars, and do not seem to conform to an overriding grammatical structure, forcing one commentary to conclude that “the emperor of UG has no clothes” (Evans and Levinson, 2009).

In his preface to the most recent edition of *The Minimalist Program*, though, Chomsky (2015) makes clear his view that UG is fundamentally a property, not of communicative language itself, but rather of thought, and is only incidental to communication. He writes: “It is a familiar fact (*sic*) that the complexity and variety of language appears to be localized overwhelmingly—and perhaps completely—in externalization (p. xi),” where “externalization” refers to the formation of specific languages from the underlying I-language. By extricating UG from communicative language itself, Chomsky appears to have sidestepped the problem of linguistic diversity. He also suggests that UG arose in a narrow window of time, shortly before the exodus of our species from Africa 50,000 to 80,000 years ago—a view endorsed by a number of anthropologists (e.g., Hoffecker, 2007; Tattersall, 2012). Chomsky (2010) even suggests that the emergence of the operation merge occurred in a single individual whom he whimsically names “Prometheus.”

By reducing the essence of UG to the single operation of merge, Berwick and Chomsky (2016) claim also to have overcome the seemingly intractable problem of how a faculty as complex as language could have evolved in a single step, in defiance of Darwinian evolution. As they put it, “… narrowly focusing the phenotype in this way greatly eases the explanatory burden for evolutionary theory—we simply don’t have as much to explain, reducing the Darwinian paradox” (p. 11). They go on to write, though, that “Any residue of principles of language not reducible to Merge will have to be accounted for by some other evolutionary processes—one that we are unlikely to learn much about, at least by presently understood methods …” (p. 71); and they insist that “there is no room in this picture for any precursor to language” (p. 71).

My suggestion here, though, is that the recursive, generative nature of language may reside, not in a specialized I-language or UG, but in mental time travel itself, or more generally in our capacity to entertain thoughts not tied to the present. Such thoughts are the essence of imagination, defined by the Merriam-Webster Dictionary as “the act or power of forming a mental *image* of something not present to the senses or never before wholly perceived in reality.” Imaginative thoughts carry the generativity and recursiveness exemplified in our reconstructions of the past, in mental anticipations of the future, and perhaps most commonly in the fabrication of stories (McBride, 2014; Boyd, 2009). In providing the means to communicate such events, language requires the property of *displacement*, the capacity to refer to the non-present (Hockett, 1960), and arguably the most important driver of its evolution. Again, though, this capacity may reside not in language itself, but rather in the imaginative construction of mental events.

## Uniquely Human?

Tulving (2002) view on the emergence of episodic memory echoes Chomsky’s account of the late arrival of UG itself:

*Many nonhuman animals, especially mammals and birds, possess well-developed knowledge-of-the-world (declarative, or semantic, memory) systems and are capable of acquiring vast amounts of flexibly expressible information. Early humans were like these animals, but at some point in human evolution, possibly rather recently, episodic memory emerged as an “embellishment” of the semantic memory system (p. 7)*.

By extension, mental time travel has also been attributed uniquely to humans and denied to all other species (Suddendorf and Corballis, 1997, 2007).

More recently, I have argued that, on the contrary, the origins of mental time travel may go far back in evolution (Corballis, 2013; but see also Suddendorf, 2013). This change of opinion is based partly on behavioral evidence for mental time travel in a diverse range of species, including great apes (Martin-Ordas et al., 2010; Beran et al., 2012; Janmaat et al., 2014), meadow voles (Ferkin et al., 2008), rats (Wilson et al., 2013), ravens (Kabadayi and Osvath, 2017), scrub jays (Clayton et al., 2003), and even cuttlefish (Jozet-Alves et al., 2013). In one recent study, rats remembered many different episodes over intervals of up to 45 min without any evidence of decline in performance (Panoz-Brown et al., 2016).

### Role of the Hippocampus

Evidence also comes from neuroscience, much of it focused on the hippocampus, and on parallels between human and animal hippocampal function. In humans, the hippocampus plays a critical role in declarative memory, including episodic memory and its extension to episodic future thinking. People with destruction of the hippocampus show striking difficulties in recalling past events or imaging future ones (Tulving, 2002; Wearing, 2005; Corkin, 2013), as well as in imagining fictitious scenes (Hassabis et al., 2007)—although impairment of the ability to imagine personal past or future events has also been linked to damage of the ventromedial prefrontal cortex (Bertossi et al., 2016).

Brain imaging confirms the role of the hippocampus when people are asked to recall previous episodes or to imagine future ones (Addis et al., 2011; Martin et al., 2011). Again, though, areas other than the hippocampus are also active, including the angular gyrus, the medial frontal cortex, and the posterior cingulate (Rugg and Vilberg, 2013; Karapanagiotidis et al., 2017). The particular role of the hippocampus may lie in what has been termed *scene construction* (Maguire et al., 2016), the drawing together of dispersed information for autonoetic inspection. McCormick et al. (2018) suggest that hippocampal function goes beyond mental time travel to mind-wandering more generally, and lies at “the heart of mental life” (p. 2745).

In the rat, the hippocampus is well known to play a role in spatial location. So-called “place cells” record the animal’s location in space, creating a “cognitive map” (O’Keefe and Nadel, 1978)—or a kind of internal GPS system. The population of active cells shifts as the animal moves around, recording a trajectory. It has become clear, though, that the activity of place cells is not restricted to the present, but can convey information about past trajectories or even trajectories that it did not take, perhaps representing future plans or simply exploratory movements. Such trajectories have been described as “replays,” although in many cases they might be better described as preplays or mental explorations not specifically located in time. Reviewing the evidence, Moser et al. (2015) write that:

*“the replay phenomenon may support ‘mental time travel’ … through the spatial map, both forward and backward in time (p. 6)*.”

Hippocampal activity, in conjunction with the neighboring entorhinal cortex, is also tagged in other memory-like ways. Place cells respond not only to specific locations, but also to nonspatial features of past events, such as odors (Igarashi et al., 2014), touch sensations, and the timing of events. Similar associations seem to be tagged to place cells in the human hippocampus. In one study, human patients about to undergo surgery had electrodes implanted in cells in the medial temporal lobe, in an attempt to locate the source of epileptic seizures. They were given the task of navigating a virtual town on a computer screen and delivering items to one of the stores in town. They were then asked to recall only the items and not the location to which they were delivered. The act of recall, though, activated place cells corresponding to that location, effectively mirroring the replay of place-cell activity in the rat brain (Miller et al., 2013). In a similar study using subdural electrodes, Vaz et al. (2019) found that oscillatory activity between the medial temporal lobe and the temporal associative cortex were coupled when people retrieved memories of associated items.

The spatial function of the hippocampus is modulated by activity in the neighboring entorhinal cortex. So-called grid cells in the medial entorhinal cortex code locations corresponding to spatial features such as spatial scale and orientation, and other cells code shape and color, proximity to borders, and direction in which the head is facing (Diehl et al., 2017). These cells operate in a modular fashion, creating an enormous number of combinations reflecting the possible spatial contexts in which an animal may find itself. Moser et al. (2015) liken this to “an alphabet in which all words of a language can be generated by combining only 30 letters or less” (p. 11). This is suggestive of the generativity of language itself.

Recordings from the rat hippocampus also reveal what has been termed “time cells,” which respond in a coordinated fashion to code the relative times in which events have occurred in the past. The pattern itself changes over time as the temporal context changes (Eichenbaum, 2017). This can be observed experientially in our own memories of when things happened, gradually losing immediacy and detail, both spatial and temporal. The hippocampal coding of space, time and context in both humans and animals suggest that episodic mental travel may long predate human evolution.

The coding of episodic memories can be specified in time rather than space, and need not be visual. We might mentally replay a memory of a concert, but the ordering of individual pieces is not marked by different locations. A similar phenomenon has been reported in rats, based on their fine discrimination of different odors. Panoz-Brown et al. (2018) presented rats with sequences of specific odors in different contexts. Later, when presented with one given context, they were able to select the second from the last odor in the sequence as distinct from a different odor from the sequence, while given a different context they were able to select the fourth from the last odor in the sequence. The number of odors in the sequences varied from trial to trial, making it impossible to specify the required odor when it occurred. The animals must have held the entire sequence in memory and replayed it in order to select the required odor. Performance was well above chance even after the lapse of an hour between presentation and test and was little affected by interference. Performance dropped significantly with chemical suppression of hippocampal activity. These properties imply robust hippocampal-dependent episodic memory for sequences of events defined by the order in which they occurred and not by locations within sequences, although the retrieval of the sequences themselves depended on spatial context.

The evidence for mental time travels in nonhuman animal raises the question of whether they are conscious. To Tulving, episodic memories are what he called *autonoetic*, or part of personal consciousness, and the same might be said of mental time travel more generally. If such travels are not exclusive to humans, contrary to what Tulving believed, can we conclude that animals too are conscious of their mental time travels? The commonality between what we know of the role of the hippocampus through electrophysiology in animals and through brain imaging, and indeed through cases of implanted electrodes in humans, seems to give little reason to doubt that in both cases the experience is conscious. Nevertheless, this is likely to remain a contentious issue.

The role of the hippocampus is not restricted to episodic information, but includes semantic information as well—indeed the replay of the past and prediction of the future is probably always a mix of the episodic and the semantic (Klein, 2013). Duff and Brown-Schmidt (2012) review evidence from studies of hippocampal amnesia that the hippocampus is critical to language itself, in binding information from different sources and supplying a flexibility of operation. Piai et al. (2016) add evidence from recording of hippocampal theta during sentence processing, and suggest that the hippocampus should be considered part of the language network, a conclusion endorsed by Covington and Duff (2016). Individuals with large-scale destruction of the hippocampus can retain the basic ability to speak, but loss of episodic memory, and of mental time travel more generally, severely restricts communicative content (Wearing, 2005; Corkin, 2013), and word learning becomes sparse and slow (Warren and Duff, 2019). The hippocampus not only contributes to the generative and integrative aspects of language, but also provides for displacement, the power of language to refer to events removed from the present in time and space.

### Expansions of Scale

A good deal of human language has to do with events or material far displaced from the present; we can tell of events from childhood, experiences in far-away places, or plans for a distant future. This suggests that mental time travel itself may have expanded in scale beyond that evident in other species, and indeed this expansion may have partly driven the evolution of language itself–although such a claim may well simply reflect what has been called the “human superiority complex” (Villa and Roebroeks, 2014, p. 1). Many animals and birds do appear to have extensive understanding of space. Dolins et al. (2014) assessed the ability of humans and chimpanzees to learn complex virtual environments and navigate through them, and found chimpanzees generally on the same level as children, and one chimpanzee (Panzee) was more accurate than human adults. Nevertheless, it is likely that the capacity for mental excursions probably expanded in both time and space, and indeed content, over the past six million years or so with the emergence of the hominins, and especially the genus *Homo*. Humans probably have more extensive memories, plans, and fantasies than do rats and chimpanzees.

In human evolution, a critical period for such an expansion, and indeed for the pressure to communicate about it, was probably the Pleistocene, dating from some 2.8 million to 12,000 years ago, when our forebears adapted to a post-arboreal existence, with an emergent hunter-gatherer pattern. This resulted in long delays between the acquisition and the use of tools, as well as geographical distance between the sources of raw material for tools and killing or butchering sites (Gärdenfors and Osvath, 2010). The hunter-gatherer lifestyle involved frequent shifts of camp as resources were depleted, forcing the group to move on to another more abundant region—a pattern still evident in present-day hunter-gatherers (Venkataraman et al., 2017).

Migrations increased in scale during the Pleistocene, adding further to the demands of space, time, memory, and planning, and brain size also tripled during this era (Klein, 2009). The dispersals of early *Homo* from Africa reached the Loess Plateau in China by 2.1 million years ago (Zhu et al., 2018), and other widespread regions in Europe and Asia in the previous millennium (Kappelman, 2018). Later waves of migration of *Homo sapiens* out of Africa began from about 120,000 years ago (Timmermann and Friedrich, 2016), eventually inhabiting most of the globe. Of course, humans are not entirely alone in undertaking large-scale migrations. Birds, whales, wildebeest, and even butterflies migrate vast distances, but these are largely seasonal (as are some human migrations, especially of the wealthy) and based on instinct rather than planning. The Clark’s nutcracker is said to cache some 33,000 seeds in around 7,000 locations every fall and relies on spatial memory to recover them over the winter (Kamil and Balda, 1985). Evidence from scrub jays, moreover, suggests that caching behavior involves mental time travel both forward and backward in time (Clayton et al., 2003). Even so, the human ability to recapture the past and imagine the future, at least with respect to time and flexibility, probably exceeds that of any other living animal.

Perhaps the ultimate stretch is the ability to imagine events outside of the lifespan, although this is a matter of semantic rather than episodic time travel—or what Klein et al. (2010) call *known time* as distinct from *lived time*. Historical records have allowed us to create stories and movies reconstructing events long in the past, and even to imagine ourselves as spectators. Physicists have even dared to envisage the origins of the universe. We also imagine life after death. Pettitt (2018) notes that even chimpanzees follow certain *mortuary behaviors* on finding a dead conspecific, including staying by the body for many hours, giving alarm calls, and showing signs of grief, as though aware of the permanence of death. The parallels with observation of human reactions to death, he suggests, “are striking” (p. 6). In humans, this is further transformed into burial and rituals associated with it, and in the modern world most of these rituals seem to have to do with “transforming the deceased into some form of afterlife” (p. 6). Evidence for the deliberate disposal of corpses, implying a sense of one’s own mortality, has been dated from around 600,000 to 300,000 years ago (Egeland et al., 2018).

## Communication

These expansions in time and space no doubt added to the pressure to communicate, so that experiences not restricted to the immediate environment could be shared, and indeed make up much of what we call culture. Communication of our internal thoughts is what Chomsky (2015) called *externalization*. Again, the critical period of its development was probably the Pleistocene. Beginning with the hunter-gatherer phase, and extending to more complex modes of existence through the development of farming and manufacture, life depended increasingly on cooperation and the sharing of experience, plans, and mental exploration leading to stories. The main requirement for communicating internal mental events, though, was a signaling device capable of matching the generativity and complexity of experience itself. Most animals have only a limited range of systems permitting intentional output. Neurophysiology increasingly reveals the complexity of the rat’s excursions in time and space, but the animal has no obvious way to convey those excursions to others. Songbirds are something of an exception, with often complex songs, but these seem adapted to sexual or identification signaling rather than to the sharing of memories or plans. They appear not well adapted to communicating about events.

Even non-human primates have very limited vocal repertoires, dedicated for the most past to instinctive or emotional calls. Seyfarth and Cheney (2018) identify different baboon calls signaling identity, social rank, kin and various social interactions, and go so far as to suggest that the sequences of calls between different individuals constitutes a system that is “*discrete, combinatorial, and rule-governed*” (p. 28, their italics), with the implication that it may be a precursor to grammatical language itself. But as Godfrey Smith (2018) points out, the combinatorial structure is evident in the interweaving of calls between individuals, and not in individual calls themselves.

The problem of communication is largely one of production rather than reception. The understanding of spoken words can actually be quite high in nonhuman animals. Border collies have been shown to respond to verbal requests to select a particular object from an otherwise uninhabited room and returns it to a given location. One border collie called Rico has a receptive vocabulary of over 200 items (Kaminski et al., 2004), while another is said to respond to the names of 1,022 objects (Pilley and Reid, 2011). Kanzi, a bonobo, appears able to respond appropriately to simple spoken requests, such as “Could you carry the television outdoors?” or “Could you put the pine needles in the refrigerator?” (Savage-Rumbaugh et al., 1998).

None of these species, though, can speak. A fundamental problem is that most mammals and apes, with the exception of humans, have at best limited voluntary control over voicing. Chimpanzees seem to have some ability to modify emotional calls (e.g., Slocombe and Zuberbühler, 2005) but little evident capacity to produce or learn anything like spoken words, either in number or complexity. According to Petkov and Jarvis (2012), only parrots approach humans as “high vocal learners,” with songbirds not far behind, while nonhuman primates are merely “limited vocal learners.” The origins of communicative language may lie in the production of visual signals, rather than vocal ones (Corballis, 2017).

Chimpanzees and bonobos trained to use lexigrams to refer to objects and actions are able to use these, along with gestures, to make requests and even to comment on past and future events, or on other individuals (Lyn et al., 2011). In one study, the chimpanzee Panzee, who uses a keyboard containing 256 lexigrams, watched an experimenter hide objects in the woods outside her enclosure. After imposed delays of up to 16 h, she interacted with a person who did not know that an object had been hidden, pointed to the lexigram representing the object, pointed outdoors, and led the person to where the object was hidden, continually pointing as she went (Menzel, 1999). There were 34 such trials, with different objects and locations. Panzee, therefore, seems capable not only of mental time travel, but also of displacement in her ability to communicate.

Chimpanzees in the wild gesture prolifically to each other, in an intentional fashion. Byrne et al. (2017) report evidence for repertoires of at least 66 natural gestures in the chimpanzee, 68 in bonobos, 102 in gorillas, and 64 in orangutans, considerably larger than repertoires of vocal calls. Many of those observed in the wild are common to the different species, suggesting that they are based on phylogeny rather than social learning, but they are also greatly augmented in the case of apes trained to use gestures or lexigrams. The gorilla Koko, for example, is said to use and understand over 1,000 signs (Patterson and Gordon, 2001).

Gestures are also more obviously intentional than are vocal calls, and are in that sense language-like, but they are more deictic than referential (Byrne et al., 2017), and occur in short sequences of seldom more than one or two, with no evidence of syntactic structure.

The ability to generate complex sequences probably emerged in human evolution with pressure to communicate about more complex events or plans. Given our ape physiognomy, a natural way to communicate mental time travels would be through pantomime, and apes do seem capable of limited pantomime. Russon and Andrews (2001) identified 18 different pantomimes produced by orangutans in a forest-living enclave in Indonesia, 14 addressed to humans and four to fellow orangutans. These included mimed offers of fruit, enacting a haircut, and requests to have their stomachs scratched by scratching their own stomachs and then offering a stick to the prospective scratcher. A chimpanzee in the wild watched her daughter trying to use a stone to crack a nut and then enacted the operation to show her how to do it properly (Boesch, 1993). Tanner and Perlman (2017) also note that gorillas combine gestures in sequence creatively and interactively, although this seems to have more to do with play and personal display than with propositional communication, and may be the origin of music and dance rather than of language itself. Nevertheless, it seems likely that language did emerge from primate gestures rather than vocal calls. Based on studies of gestural communication in apes, Tomasello (2008, p. 55) refers to gestures as “the original font from which the richness and complexities of human communication and language have flowed.”

But it was probably during the Pleistocene, with the so-called “cognitive niche” (Pinker, 2010) as an adaptation to the more dangerous and uncertain environment, that gesture, perhaps originally as pantomime, emerged as a powerful way to share episodic events, whether past, future, or simply invented. Donald (1991) referred to the “mimetic culture” of the early Pleistocene. Pantomime involves whole-body action to represent events, but the essence of an event in space and time could be relayed more economically just using gestures of the hands and arms, which were freed from any involvement in locomotion with the advent of bipedalism. Gestural language may well have developed to resemble modern sign languages invented by deaf communities. Emerging sign languages typically begin with pantomime, but signs are then conventionalized so that many no longer provide a pictorial indication of what they stand for (Burling, 1999). Conventionalization may be at the cost of transparency but leads to greater efficiency. On an evolutionary scale, speech itself may be the end product of a conventionalization process that began with pantomime, as our forebears gained great intentional control over voicing.

Nevertheless, bodily gesture remains an integral accompaniment to speech, even in the blind (Iverson and Goldin-Meadow, 1998). They can improve the speaker’s lexical access and fluency (Rauscher et al., 1996), and even reduce the speaker’s working memory load (Goldin-Meadow et al., 2001; Wagner et al., 2004). Some have gone so far as to suggest that manual gestures were in equal partnership with vocalization throughout the evolution of language (e.g., Kendon, 2011; McNeill, 2012), but the evidence from primates suggests that manual gestures preceded vocalization in the evolution of intentional communication (Corballis, 2014). It remains something of an open question when speech evolved to the level of articulation evident in *Homo sapiens*. It is possible, even likely, that the one of our closest forebears, the Neanderthals, were capable of speech (Dediu and Levinson, 2013), but their articulation was probably relatively more restricted through non-optimal development of the vocal tract (Gokhman et al., 2019).

## Conclusions

The main thesis of this article is that imagination, initially in the form of mental travels in time and space, provide the recursive and generative properties underlying language itself. These mental travels are extensions of episodic memory and make up much of what we call imagination. Unlike Chomsky’s concept of I-language, imagination probably has a long evolutionary history, as is becoming evident from behavioral studies in a wide variety species, along with work on the role of the hippocampus and related structures in rodents. Communicative language is then the externalization of imagination, and different languages use different conventions to express the products of imagination. This approach differs from that of Chomsky and colleagues also in that both imagination and its externalization have a strong evolutionary basis in spatial understanding and bodily movement, whereas I-language is regarded as abstract and symbolic, creating what is known as the problem of *grounding* (Harnad, 1990). How can a person relate abstract symbols to events in the real world?

In the view adopted here, symbolic representation arises in the process of externalization, rather than in innate symbolic dispositions. Internal representations of objects, actions and events are for the most part similar across different peoples (corresponding to the “universal” of UG), but the symbols to represent them differ markedly. Some 6,000 languages exist in the world, each more or less incomprehensible to almost every other. As suggested earlier, those symbols probably begin as iconic, or pantomimic, but become increasingly arbitrary and abstract in the process of conventionalization. This process increases efficiency, but may also be driven by exclusiveness, acting as a barricade to outsiders. Language operates in part as a secret code. Sign languages are more transparent, but even they conventionalize differently. Nevertheless, it is also becoming clear that many speech sounds are non-arbitrary, and show similar associations across language groups (Blasi et al., 2016).

The symbols that arise in the process of externalization themselves become part of our semantic memories; we can imagine the word “dog” as easily as we can imagine the animal with which it is associated. We can play with words just as we play with toys. The use of abstract symbols may well have influenced cognition itself. Mathematics may be an extreme example, in which abstraction has developed to the point that a single symbol, say x or y, can stand for variables of wide reference. But the invention of abstract symbols was not the outcome of some singular event in our evolutionary past, but was the product of gradual evolution, perhaps leading to increased powers of reasoning and discourse.

Given that words themselves have become part of memory, the emergence of language may well have expanded our capacity for mental time travels, and perhaps especially for the imaginative extensions into fiction and storytelling. The capacity to communicate our mental excursions vastly exceeds that required for personal experience alone. To accommodate the information added through communication, including not only speech, but also the vast repertoire of information through books, films, television, and so forth, storage capacity itself must surely have expanded. The link between language and memory might, therefore, be considered bidirectional.

From the Bible to Chomsky, the emergence of language has been regarded as a singular event, bestowed uniquely on our own species. The concept of time, too, is also widely viewed as uniquely human. Donald (1991), for example, wrote that “The lives of apes are lived entirely in the present” (p. 149), and much earlier Kohler (1927), based on his studies of problem solving in chimpanzees, wrote that “the time in which chimpanzees live is limited in past and future” (p. 272). The poet Robert Browning, in his 1885 poem “A Grammarian’s Funeral,” prophetically wrote:

“He said, What’s time? Leave Now for dogs and apes

Man has Forever!”

Contrary to these commonly held views, the experience of past and future probably goes far back in the evolution of animals that move, and need to know where they are, where they have been, and where they might go next—along with what happened or might happen there. The sharing of this information, though, probably evolved later, as our forebears were forced for survival into their cognitive niche.

One-hundred and sixty years after the publication of Darwin’s (1859) *Origin of Species*, it is time to work toward an evolutionarily plausible understanding of how the human mind evolved.

## Data Availability

All datasets analyzed for this study are included in the manuscript and the supplementary files.

## Author Contributions

The author confirms being the sole contributor of this work and has approved it for publication.

## Conflict of Interest Statement

The author declares that the research was conducted in the absence of any commercial or financial relationships that could be construed as a potential conflict of interest.
